# Effects of Lidocaine-Mediated CPEB3 Upregulation in Human Hepatocellular Carcinoma Cell Proliferation In Vitro

**DOI:** 10.1155/2018/8403157

**Published:** 2018-04-17

**Authors:** Hongjun Liu, Yiru Wang, Bing Chen, Xia Shen, Wenxian Li

**Affiliations:** Department of Anesthesiology, The Eye, Ear, Nose and Throat Hospital of Fudan University, Shanghai Medical College of Fudan University, Shanghai, China

## Abstract

Lidocaine displays antitumor activity by inducing apoptosis and suppressing tumor growth in human hepatocellular carcinoma (HepG2) cells in vitro. However, the molecular mechanism underlying lidocaine-mediated antitumor activity is unclear. In this study, HepG2 cells were treated with lidocaine, and cell proliferation and colony-forming ability were assessed. The expression level of cytoplasmic polyadenylation element binding protein 3* (CPEB3)* was detected by real-time quantitative PCR and western blot. Lidocaine treatment resulted in decreased HepG2 cell viability and colony formation in a dose-dependent manner. In hepatocellular carcinoma patient samples, CPEB3 was downregulated and was associated with poor prognosis and high-grade malignancy. Additionally, CPEB3 was a critical mediator of lidocaine-induced repression of HepG2 cell proliferation. These results demonstrated that lidocaine decreased cell viability and colony-forming ability of HepG2 cells by upregulating CPEB3 expression.

## 1. Introduction

Hepatocellular carcinoma (HCC) is one of the most common malignancies in the world and is associated with poor patient prognosis [1]. According to the latest* Global Cancer Statistics*, HCC is the fifth most prevalent cancer in men and the ninth most prevalent cancer in women. In 2010, there were approximately 782,500 new cases of HCC detected globally and approximately 745,500 HCC-related deaths [2]. Surgery remains an important treatment option for HCC [3]. However, other complicating factors (e.g., perioperative care and anesthetic management) may influence disease progression and recurrence [4, 5]. Several retrospective studies have reported that regional anesthesia, epidural analgesia, and perioperative analgesia could influence disease-free survival outcomes for cancer patients [6–8]. Lidocaine, a local anesthetic, is widely used for regional anesthesia and pain relief in the clinic. Previous studies have demonstrated that lidocaine effectively inhibited the proliferation and invasion of tumor cells and that lidocaine use was associated with improved disease-free survival outcomes for cancer patients after surgery [9–13]. However, the molecular mechanisms underlying this improved patient outcome remain unknown.

Cytoplasmic polyadenylation element binding protein 3 (CPEB3) is a member of the CPEBs family, which can regulate translation by modulating cytoplasmic polyadenylation. Aberrant expression of CPEB3 has been observed in several tumors. CPEB3 is downregulated in colorectal cancer [14], human HCC [15], and cervical cancer [16]. Furthermore, some microRNAs can promote tumor progression by targeting the CPEB3/estimated glomerular filtration rate (EGFR) axis [17, 18]. These findings suggested that CPEB3 could play an important role in regulating tumor progression. Although previous studies have confirmed the tumor suppression effect of local anesthetics, the potential mechanism has not been fully understood. Thus, we hypothesized that lidocaine may confer a protective antitumor effect through the upregulation of CPEB3.

## 2. Materials and Methods

### 2.1. Cell Culture and Key Reagents

Human HCC-derived HepG2 cells were kindly provided by the Stem Cell Bank at the Chinese Academy of Sciences (Shanghai, China). The cells were cultured in high glucose Dulbecco Modified Eagle medium (DMEM) (Gibco, New York, USA) containing 10% (v/v) fetal bovine serum (FBS) (Gibco, New York, USA) and were maintained at 37°C in a humidified incubator containing 5% CO_2_. Lidocaine hydrochloride with a purity of 2% (5 ml; 0.1 g. molecular weight = 288.82) was purchased from Hualu Pharmaceutical Co., Ltd. (Shandong, China). Lidocaine concentrations are expressed in millimolar (mM) values instead of percentages (%): 2% = 69.25 mM and 0.2888% = 10 mM.

### 2.2. Cell Viability Assay and Drug Titration

Cell viability was assessed using the Cell Counting Kit-8 (CCK-8) assay (Dojindo Molecular Technologies, Tokyo, Japan) following the manufacturer's instructions. Briefly, the HepG2 cells were seeded into 96-well plates (3,000 cells per well) and treated with 100 *μ*l fresh serum-free DMEM medium containing varying concentrations of lidocaine (0, 0.1, 0.2, 0.5, 1, 2, 5, and 10 mM) for 24 hours. The CCK-8 reagent was added to each well at a volume of 10 *μ*l, and the plate was incubated for 3 hours at 37°C. The absorbance value (OD) was read at 450 nm using a spectrophotometer, and the data were analyzed using Synergy H1 Hybrid Reader (BioTek, Vermont, USA). Survival rates were calculated following the manufacturer's instructions of CCK-8 ([(As-Ab) − (Ac-Ab)] × 100% where As is experimental group; Ac is conditional control group; Ab is untreated control group). Each experiment was performed in triplicate.

### 2.3. Colony Formation Assay

The HepG2 cells were seeded on a six-well tissue culture plate (1,000 cells per well). Cells were treated with the indicated concentrations of lidocaine (0, 1, 2, and 5 mM). Cells were stained with 0.5% crystal violet (in methanol) and the colonies were counted 14 days later. Colony formation assay was performed in triplicate at each drug concentration.

### 2.4. Quantitative-Polymerase Chain Reaction (qPCR) Analysis

The HepG2 cells were seeded into T25 culture flask and were left untreated or treated with 2.5 mM lidocaine for 24 hours. Total RNA was extracted with TRIzol reagents (Invitrogen, Carlsbad, USA) according to the manufacturer's instructions and quantified by spectrophotometry at a wavelength of 260 nm. Subsequently, 2,500 ng RNA was reverse-transcribed into complementary DNA (cDNA) in a 50 *μ*l reaction using the RevertAid First Strand cDNA Synthesis kit (ThermoFisher Scientific Inc., MA, USA). qPCR was performed in a ViiA7 Real-Time PCR machine (Applied Biosystems by ThermoFisher Scientific Inc., MA, USA). The primers used were as follows: CPEB3 forward, 5′-GAGCTGTTGAACTGGCAATG-3′ and reverse, 5′-ACTGCAGACAGGTGACGTTG-3′; and GAPDH (reference gene) forward, 5′-CGGAGTCAACGGATTTGGTCGTAT-3′ and reverse, 5′-AGCCTTCTCCATGGTGGTGAAGAC-3′. The reaction was initiated with denaturation at 95°C for 30 seconds; followed by 40 cycles at 95°C for 5 seconds and 60°C for 30 seconds (annealing); and 95°C for 15 seconds (terminal extension step) followed by a final holding step at 4°C. Relative CPEB3 mRNA expression was defined as the ratio of CPEB3 gene expression to GAPDH expression. The experiment was repeated three times.

### 2.5. Western Blot

Cells were lysed in ice-cold radioimmunoprecipitation assay (RIPA) buffer (Beyotime Biotechnology, Shanghai, China) for total protein extraction. Total protein concentrations were calculated using the BCA Protein Assay kit (Vigorous Biotechnology, Beijing, China). Proteins were then separated by sodium dodecyl sulfate-polyacrylamide gel electrophoresis (SDS-PAGE) and transferred to polyvinylidene difluoride (PVDF) membranes. The membrane was blocked for 1 hour with 5% nonfat milk at room temperature and incubated with the CPEB3 antibody (Novus Biologicals, Colorado, USA, 1 : 1000) or *β*-actin antibody (Beyotime Biotechnology, Shanghai, China, 1 : 1000). After incubation with secondary antibodies (horseradish peroxidase-conjugated anti-rabbit IgG, 1 : 2000) (Proteintech, Hubei, China), the proteins of interest were detected using the enhanced chemiluminescence system (Pierce Biotechnology, MA, USA).

### 2.6. CPEB3 Knockdown and Cell Viability Assessment

The CPEB3 short hairpin RNA (shRNA) plasmid was purchased from GeneChem Co., Ltd. (Shanghai, China). The lentiviral expression vector GV248 was used for expressing the shRNA. The shRNA target sequence (TCCTTAATGGATATGATAA) was selected for* CPEB3* downregulation. The nontargeting sequence (TTCTCCGAACGTGTCACG) was used as a negative control. Oligomeric single-stranded DNA for the shRNA was designed and synthesized according to these gene sequences. The oligomeric single-stranded DNA was annealed to obtain a double-stranded shRNA, which was then inserted into the shRNA lentiviral vector to make the reconstructed shRNA lentiviral plasmid. Competent* E. coli* TOP10 cells were transfected. The HepG2 cells were transfected with* E. coli* TOP10-EGFP-shRNA using Lipofectamine 3000 (ThermoFisher Scientific Inc., MA, USA) to knock down the* CPEB3* gene. The transfection efficiency was evaluated by measuring the green fluorescence in the cells using microscopy and flow cytometry. After knockdown of the* CPEB3* gene was confirmed, we repeated the CCK-8 cell viability assay to investigate the effect of lidocaine on CPEB3-knockdown cells.

### 2.7. TCGA Dataset Analysis

We searched the cancer genome atlas (TCGA) database (https://cancergenome.nih.gov/) and obtained the whole genome mRNA expression data from HCC samples. We used the RSEM software package for downstream analysis.* t*-test was used to compute whether* CPEB3* expression differences between tumor samples and normal samples were significant. For survival analysis, patients were separated into two groups according to the median expression level of* CPEB3*. We also compared the expression levels of* CPEB3* in neoplasms with different histological grades according to the 4-scale Edmondson and Steiner system.

### 2.8. Statistical Analysis

Statistical analysis was performed using GraphPad Prism 7 (GraphPad Software, San Diego, USA). All data are presented as the mean ± standard deviation. Student's *t*-test was used for CPEB3 expression between two groups. One-way analysis of variance (ANOVA) followed by a Bonferroni post hoc test was applied for cell survival rate and CPEB3 expression among different groups. *P* < 0.05 was considered a statistically significant difference.

## 3. Results

### 3.1. Lidocaine Inhibited the Proliferation of HepG2 Cells

**Figure 1:**
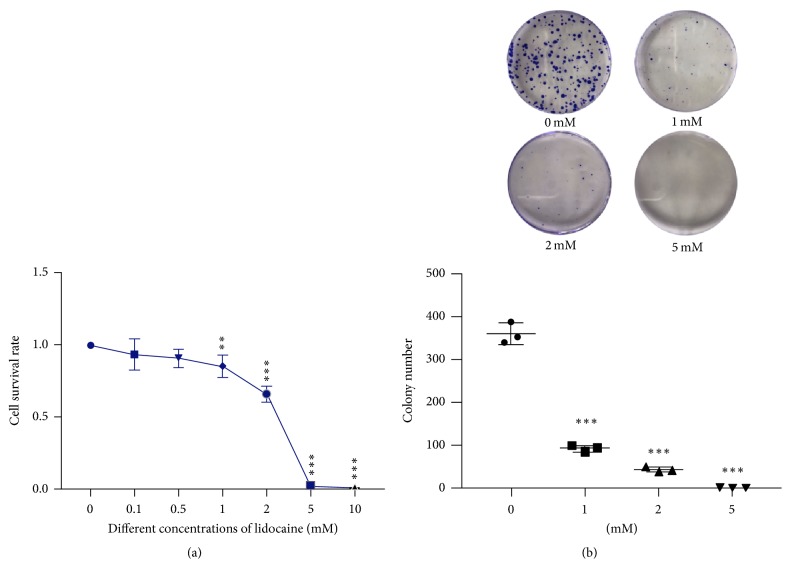
Lidocaine inhibited HepG2 cell proliferation. (a) Lidocaine suppressed HepG2 cell viability in an approximately dose-dependent manner. (b) Lidocaine reduced HepG2 cell colony-forming ability in a dose-dependent manner. Compared with control untreated group, *∗∗* indicates *P* < 0.001; *∗∗∗* indicates *P* < 0.0001. *n* = 3 per drug concentration.

Lidocaine decreased HepG2 cell viability in an approximately dose-dependent manner as detected by the CCK-8 Assay. Treatment with 1, 2, or 5 mM lidocaine for 24 hours resulted in significant reductions in cell viability (*P* < 0.001; *n* = 3). Treatment with a high concentration of lidocaine (10 mM) resulted in complete loss of HepG2 cell viability (*P* < 0.001; *n* = 3). Lower concentrations of lidocaine (0.1 or 0.5 mM) had no effect (*P* > 0.05; *n* = 3; Figure 1(a)). Furthermore, lidocaine inhibited HepG2 colony-forming ability in a dose-dependent manner. While untreated HepG2 cells formed 362 ± 26 colonies, lidocaine treatment resulted in 94 ± 8 colonies at a concentration of 1 mM (*P* < 0.001), 44 ± 6 colonies at a concentration of 2 mM (*P* < 0.001), and no colonies at a concentration of 5 mM (*P* < 0.001) (Figure 1(b)).

### 3.2. Lidocaine Upregulated CPEB3 mRNA and Protein Expression

**Figure 2:**
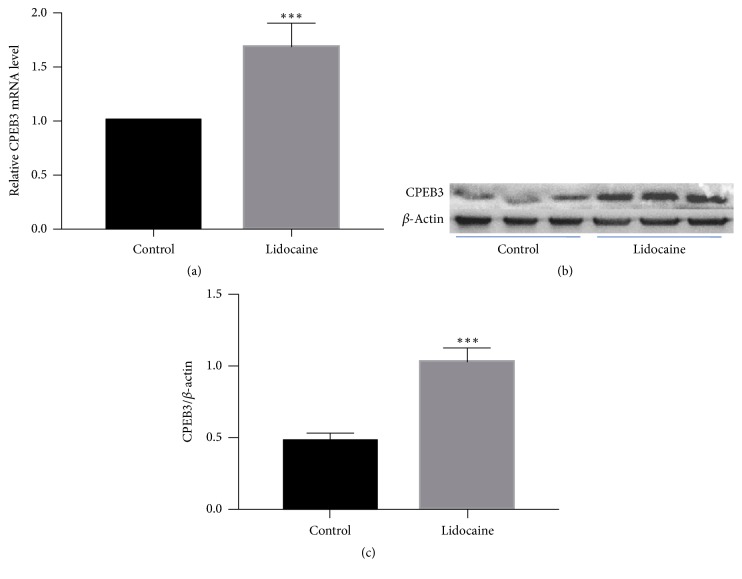
Lidocaine (2.5 mM) upregulated CPEB3 expression in HepG2 cells. (a) Compared to the control untreated group, the expression of* CPEB3* mRNA in the HepG2 cells was 1.67-fold higher after 24-hour lidocaine treatment. (b) Representative western blot images of CPEB3 and *β*-actin in tumor cells from the lidocaine and control untreated groups. (c) Quantification of the western blot confirmed CPEB3 upregulation at the protein level in tumor cells treated with lidocaine. *∗∗∗* indicates *P* < 0.0001.

Based on the cell survival rates at varying concentrations of lidocaine (Figure 1(a)), we chose 2.5 mM as the best-fit concentration of intervention (ED_50_). Compared with the untreated control group, the expression of* CPEB3* mRNA in the HepG2 cells in the lidocaine-treated group was 1.67-fold higher after 24 hours (*P* < 0.001, Figure 2(a)). Western blot confirmed that lidocaine significantly increased the level of CPEB3 protein in tumor cells (*P* < 0.05, Figure 2(b)).

### 3.3. Lower Expression of CPEB3 Was Associated with Poor HCC Patient Prognosis

**Figure 3:**
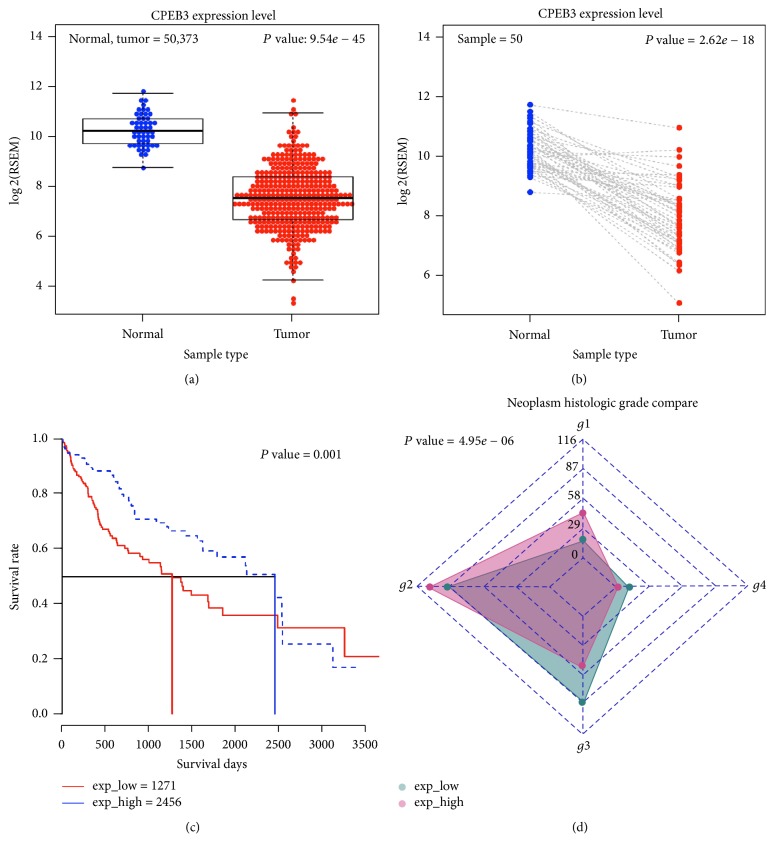
Low expression of CPEB3 was associated with poor HCC patient prognosis. (a) Expression of* CPEB3* was downregulated in tumor samples from HCC patients compared with normal samples. (b) Expression of CPEB3 was downregulated in tumor samples from HCC patients compared with paired normal samples. (c) Lower CPEB3 expression level was associated with decreased patient survival. (The red line indicated patients with lower expression level of CPEB3 while the blue line indicated patients with higher expression level of CPEB3). (d) Gene set enrichment analysis plots showed that lower expression of CPEB3 was associated with higher histological grade.

We analyzed* CPEB3* mRNA data from the TCGA database to compare expression levels in HCC patients' tumor and normal samples.* CPEB3* was significantly downregulated in tumor samples (*P* = 9.54*e* − 45, Figure 3(a);* P* = 2.6*e* − 18, Figure 3(b)). The survival rate of HCC patients was positively correlated with* CPEB3 *expression level (*P* = 0.01, Figure 3(c)). Gene set enrichment analysis plots suggested that lower expression of* CPEB3* resulted in the higher neoplasm histological grade (*P* = 4.95*e* − 06, Figure 3(d)).

### 3.4. Lidocaine Inhibited Proliferation of HepG2 Cells by Upregulating the Expression of CPEB3

**Figure 4:**
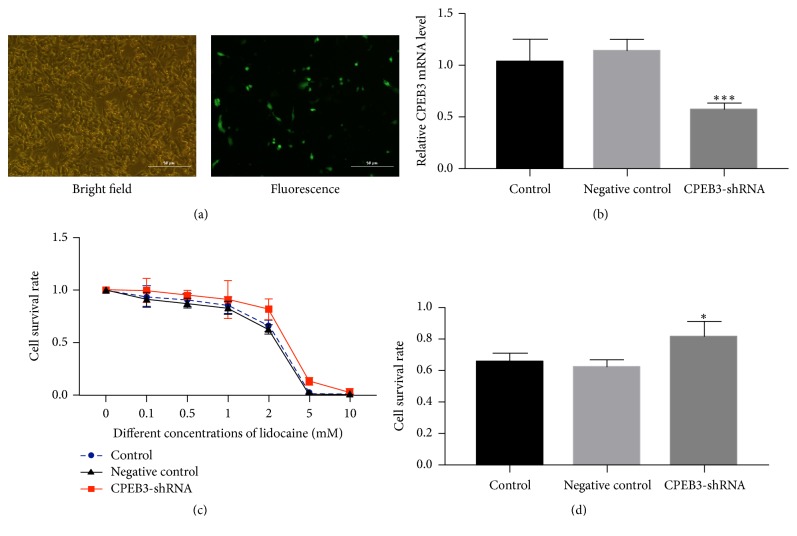
The antitumor effect of lidocaine was reversed by* CPEB3* knockdown. (a) HepG2 tumor cells were transfected with* E. coli* TOP10-EGFP-shRNA-CPEB3. Images of the same field were taken to show both the bright-field image (cell morphology) and the GFP fluorescence (100x magnification). (b) qPCR was used to quantify* CPEB3* mRNA expression in the control group (normal tumor cells), negative control (scramble shRNA), and shRNA-CPEB3 group. (c, d) HepG2 cell survival rate in shRNA-CPEB3 group after lidocaine treatment compared with control group and negative control group. Compared with control group, *∗* indicates *P* < 0.05; *∗∗∗* indicates *P* < 0.0001.

To directly test the effect of* CPEB3* expression on HCC cell survival, HepG2 cells were transfected with* E. coli* TOP10-EGFP-shRNA. On the second day posttransfection, HepG2 tumor cells exhibited robust green fluorescence (Figure 4(a)). In addition, compared with control group (normal tumor cells) and negative control group (scramble shRNA), mRNA expression of CPEB3 was demonstrated to be downregulated following transfection with* E. coli* TOP10-EGFP-shRNA-CPEB3 even after lidocaine treatment (Figure 4(b)). Next, after 24-hour treatment with 2 mM lidocaine, the survival rate of HepG2 cells was much higher in the shRNA-CPEB3 group than control group and negative control group, suggesting that the antitumor effect conferred by lidocaine was reversed in CPEB3-knockdown cells (*P* < 0.05, Figures 4(c) and 4(d)).

## 4. Discussion

Surgery remains the primary treatment option for HCC [3], but the disease-free survival outcomes for patients remains poor even after complete resection. Perioperative care and anesthetic management, especially regional anesthesia, have been previously suggested to improve the outcomes of surgery [7, 8, 19]. Recent studies have demonstrated that anesthetics (e.g., lidocaine and ropivacaine) exerted antitumor effects by suppressing cell proliferation, inducing apoptosis, and by inhibiting cell migration [9, 10, 20–22]. Lidocaine blocked lung adenocarcinoma tumor cell invasion at clinically relevant concentrations in vitro [9]. Additionally, lidocaine administered topically within the oral cavity for cancer pain relief suppressed the proliferation of human tongue cancer cells [10]. Lidocaine has also been shown to induce apoptosis of breast tumor cells when treated with concentrations similar to those used clinically [20]. In our study, we found that lidocaine decreased cell viability and colony-forming ability of HepG2 cells in a dose-dependent manner in vitro. This finding corroborates results from a previous study in which lidocaine decreased cell viability and the colony-forming ability of human thyroid cancer cells in a dose-dependent manner [21].

Intra-arterial administration of lidocaine provides ideal pain management during the transarterial chemoembolization (TACE) procedure used for HCC management [23]. Besides, lidocaine enhanced sensitization of cancer cells to chemotherapeutic agents [21–24], but the mechanism underlying this sensitization is poorly understood. Gene expression microarrays and bioinformatics analysis of patient samples from TCGA provide new insight into the detection and treatment of HCC. CPEB3 is a member of the class of sequence-specific RNA-binding proteins, which are important in the elongation of mRNA poly(A) tails and in the regulation of polyadenylation-induced translation [25, 26]. In our study, we found that* CPEB3* mRNA and CPEB3 protein both increased in lidocaine-treated HepG2 cells. Conversely, knockdown of* CPEB3* significantly reversed the antitumor effect of lidocaine. These results indicate that* CPEB3* might represent a novel lidocaine-induced target gene and its expression levels can be used as a potential biomarker for HCC patient stratification. These findings potentially implicate CPEB3 in regulating processes such as cellular proliferation, chromosome segregation, and cell differentiation, and should be further investigated.

Some studies have demonstrated that CPEB3 expression was downregulated in colorectal cancer [14] and in human papilloma virus- (HPV-) positive cervical cancers [16]. We found* CPEB3* expression was downregulated in tumor samples from HCC patients, indicating a potential function for CPEB3 in tumorigenesis. Indeed, higher* CPEB3* expression was associated with improved HCC patient survival. We also found that CPEB3 was downregulated in neoplasms with higher histological grade. Xing et al. found that lidocaine exerted its antitumor functions by activation the MAPK pathways [21]. Pregnane X receptor (PXR), a member of the nuclear receptor superfamily of ligand-regulated transcription factors, increased p38 MAPK phosphorylation in HepG2 cells [27]. Further studies are warranted to testify the potential mechanism that lidocaine upregulated CPEB3 mRNA by activating PXR.

In conclusion, our results implicate* CPEB3* as a target gene through which lidocaine suppressed proliferation of HepG2 cells in vitro.
